# Cyclization of *γ*-hydroxybutyric acid (GHBA) as a strategy to enhance its signal in gas chromatography analysis

**DOI:** 10.1007/s11419-025-00738-z

**Published:** 2025-09-13

**Authors:** Rafal Typek, Michal P. Dybowski, Andrzej L. Dawidowicz

**Affiliations:** Department of Chromatography, Institute of Chemical Sciences, Faculty of Chemistry, Maria Curie Sklodowska University in Lublin, 20-031 Lublin, Poland

**Keywords:** Gas chromatography (GC) signal enhancement, γ-hydroxybutyric acid (GABA), γ-butyrolactone (GBL), γ-hydroxybutyric acid (GABA) cyclization

## Abstract

**Purpose:**

The aim of this work is to investigate whether precyclization of γ-hydroxybutyric acid (GABA) allows for increasing its gas chromatography (GC) signal, and if so, is it a more effective way to increase the signal of this compound than its silylation or methylation?

**Methods:**

Gas chromatography-mass spectrometry (GC–MS) and GC with flame ionization detection (GC-FID) response to GHBA before and after silylation, methylation, and cyclization were compared. The impact of injector temperature on GHBA and γ-butyrolactone (GBL) signals was assessed. Fourier transformed infra-red spectroscopy was used to examine the formation of macromolecular derivatives in the injector.

**Results:**

GHBA shows a lower GC signal than GBL due to partial polycondensation into a non-volatile polyester in the injector. Validation data were established for GHBA after each derivatization. Silylation and methylation reduced the limit of detection (LOD) by approximately 1.5- and 1.3-fold, respectively, whereas pre-cyclization led to at least a 4.6-fold decrease in LOD.

**Conclusions:**

The present study elucidates the reasons behind the low GHBA signal observed in GC analysis and, consequently, supports the recommendation to perform pre-cyclization of this compound prior to analysis. Furthermore, the findings demonstrate that although signal enhancement of GHBA can be achieved through silylation or methylation, the most substantial increase is observed following its cyclization during sample preparation. The proposed in this paper cyclization procedure is both remarkably simple and highly effective, allowing for reliable quantification of this hydroxycarboxylic acid in a variety of matrices, including plasma, urine, wine, beer, and orange juice.

**Supplementary Information:**

The online version contains supplementary material available at 10.1007/s11419-025-00738-z.

## Introduction

γ-Hydroxybutyric acid (GHBA) and its cyclic derivative γ-butyrolactone (GBL) are substances classified as controlled in most countries [1–4]. They have gained notoriety as components of so-called “date-rape drugs” and have thus become a subject of particular interest for forensic toxicologists due to their intoxicating, euphoric, disinhibiting, and amnesic effects—effects that closely resemble those of alcohol intoxication [5–9]. In the gastrointestinal tract, GHBA undergoes partial conversion to GBL. Due to GHBA’s short half-life (20—53 min), detecting its presence in the body poses a considerable challenge for forensic toxicology. Even after administration of a high dose (approximately 75 mg/kg), detection of GHBA in blood samples collected more than 8 h post-administration, or in urine samples collected more than 12 h post-administration, is extremely difficult [10–14]. GBL, in turn, undergoes rapid hydrolysis in blood and liver to GHBA via the action of a lactonase enzyme [15], leading to the simultaneous presence of trace amounts of both GHBA and GBL in the victim’s system, regardless of whether the ingested formulation contained GHBA, GBL, or a mixture of the two. Although both compounds can be quantitatively determined using liquid chromatography-MS (LC–MS), this technique often fails to yield reliable results due to insufficient sensitivity [16–18]. The cited literature clearly supports this, particularly with regard to the analysis of GHBA/GBL at trace concentrations. As a result, researchers frequently turn to gas chromatography (GC), which not only offers greater analytical sensitivity but is also faster, more cost-effective, and easier to optimize for chromatographic separation. However, the application of GC for the detection of date rape drug components has limitations. GHBA contains both hydroxyl and carboxyl functional groups, which may undergo polycondensation in the high-temperature GC injector, forming polyesters and/or its cyclic ester, GBL [19–21]. This transformation can significantly reduce the sensitivity of GHBA detection via GC. To enhance the sensitivity of GC analysis of hydroxycarboxylic acids, these compounds are typically subjected to preliminary derivatization, most commonly silylation [22–27]. This process serves a dual purpose: it masks the polar functional groups (hydroxyl and carboxyl), thereby minimizing undesirable interactions with the stationary phase of the GC column that lead to excessive peak broadening, and it simultaneously prevents polycondensation reactions. This raises the question of whether pre-cyclization of GHBA—which results in mutual blocking of the carboxyl and hydroxyl group and thereby blocks potential polycondensation—improves its GC signal, and, if so, whether the signal intensity of the pre-cyclized compound surpasses that obtained following silylation.

It should be noted that the precyclization process of GHBA leads to its conversion into GBL. As a result, this transformation hinders the detection of GBL in the presence of GHBA. However, considering that:

- both substances are controlled in most countries,
- they undergo mutual biotransformation within the body,
- they possess very short half-lives, necessitating the use of highly sensitive analytical techniques, and
- there exists a potential for GHBA to undergo polycondensation,

it is advisable, for the purpose of confirming drug-facilitated sexual assault (DFSA), to determine the presence of GBL in biological fluids following the prior conversion of GHBA into its cyclic form. The aim of the present study is to determine whether the initial cyclization of GHBA to GBL inhibits the polycondensation process of GHBA, and if so, whether this reaction can be utilized to enhance the sensitivity of detecting the date rape drug in commercially available products and biological fluids.

## Experimental

### Materials

Acetonitrile (ACN) (LC/MS grade), *CRM* GHBA standard (1 mg/mL in methanol), *CRM* GBL standard (1 mg/mL in methanol), GBL (≥ 99%), hexamethyldisilazane (HMDS), trimethylchlorosilane (TMCS), *p*-toluenesulfonic acid (PTSA), formic acid and boron trifluoride in methanol (50%) were acquired from Merck (Merck KGaA, Darmstadt, Germany). Dichloromethane (DCM), methanol (MeOH), NaOH, H_2_SO_4_ (96%) and HCl (36%) were purchased from Avantor Performance Materials Poland (Gliwice, Poland). Helium, hydrogen, nitrogen, argon and air were obtained from Air Liquide Polska (Kraków, Poland). Deionized water was purified by the Milli-Q system (Merck Millipore, Merck KGaA, Darmstadt, Germany).

The plasma was obtained by centrifuging human blood taken from a volunteer, after obtaining his informed consent, by a professional nurse. A single closed system (Sarstedt AG, Nümbrecht, Germany) containing an S-Monovette coagulation activator (citrate 3.2%) was used for this purpose.

The urine used in the study also came from a volunteer who was informed about the purpose of its use in the research.

The all biological samples were GHBA and GBL free.

Wine, beer and orange juice were bought in local shop.

### Preparation of GHBA from GBL

Due to the unavailability of commercially pure GHBA and the need to perform a large number of experiments with its usage, it was decided to obtain GHBA from pure GBL.

In a 20 mL aqueous solution of NaOH (0.125 g/mL), 4.3 g of GBL was dissolved and heated at 40 °C with continuous stirring for 45 min. After this time, the mixture was cooled, and 6.34 mL of hydrochloric acid (36%) was added dropwise while continuously stirring. The resulting GHBA was extracted with DCM. The oily liquid obtained after evaporating the organic solvent was analyzed using HPLC to determine its purity—see Fig. 1S in supplementary materials.

### Formation of trimethylsilyl derivative of GHBA (GHBA-2TMS)

To 100 μL of GHBA solution in acetonitrile (1 mg/mL) 900 μL of silylation mixture (HMDS/TMCS/ACN 1:1:8 v/v/v) was added and heated at 35 °C for 30 min. After the reaction, the mixture was centrifuged at 8850 g for 5 min. The separated supernatant was analyzed by GC with flame ionization detection (GC-FID) to determine the degree of GHBA silylation prior to its use in subsequent experiments (see Fig. 2SA–C).

### Formation of methyl ester of GHBA (est-Me-GHBA)

The mixture composed of 200 μL of boron trifluoride in MeOH (50%) (derivatizing agent), 100 μL of methanolic solution of GHBA (1 mg/mL) and 700 μL of MeOH was heated at 35 °C for 30 min. After the reaction, the mixture was allowed to cool and, prior to its use in subsequent experiments, was analyzed by GC-FID to determine the degree of GHBA methylation (see Fig. 3SA–C).

### Formation of GBL from GHBA (cyclization of GHBA)

To determine the efficiency of the GHBA to GBL cyclization reaction in sample preparation procedure, 900 μL of PTSA solution in DCM (1 mg/mL) was mixed with 100 μL of DCM solution of GHBA (1 mg/mL). The mixture was subjected to GC-FID and GC–MS analysis. The obtained chromatograms were compared with chromatograms of GBL solution in DCM (0.083 mg/mL).

### Preparation of plasma, urine, wine, beer and orange juice samples for GC analysis of GHBA

Two types of samples were used in experiments:

- samples not fortified with GHBA, and.
- samples spiked with GHBA.

The concentration of GHBA in spiked samples of plasma, urine, wine, beer and orange juice samples was as follows: 1.0 µg/mL in plasma, beer and orange juice, 100 µg/mL in urine and 5.0 µg/mL in wine. As is known, red wine may contain trace amounts of GHBA. In order to achieve the GHBA concentration stated above for the wine, it was initially established the natural concentration of GHBA in the used wine.

#### The version without GHBA cyclization

The GHBA spiked samples of urine, wine, beer and orange juice were directly subjected to GC–MS analysis, whereas the GHBA spiked plasma samples before analysis were extracted (5 min) with DCM at volume ratio 1:1 v/v. After extraction the separated by centrifugation (980 g for 10 min) organic phase was subjected to GC–MS analysis.

#### The version with GHBA cyclization

To 1 mL of GHBA spiked sample of plasma, urine, wine, beer or orange juice 5 μL of PTSA water solution (20%) was added and vortexing for 5 min. All mixtures, except of that with plasma, were directly subjected to GC–MS analysis. In the case of plasma, the obtained mixture was extracted with DCM (1:1 v/v). The separated by centrifugation organic phase was analyzed by GC–MS.

### PTSA vs. H₂SO₄ as cyclization agents for GHBA present in plasma and water samples

#### Cyclization of GHBA using PTSA

To 800 µL of plasma or water sample containing GHBA at a concentration of 1 µg/mL, 200 µL of an aqueous PTSA solution (5 mg/mL) was added and vortex-mixed for 5 min. Each resulting mixture was then extracted with 1 mL of DCM for 5 min. Following the extraction process, the organic phases were separated by centrifugation (980 g for 10 min) and subsequently analyzed by GC–MS.

#### Cyclization of GHBA using H₂SO₄

To 800 µL of plasma or water sample containing GHBA at a concentration of 1 µg/mL, 200 µL of an aqueous H₂SO₄ solution (50%) was added and vortex-mixed for 5 min. The subsequent procedure was identical to that used for the PTSA-based cyclization.

### High performance liquid chromatography (HPLC) measurements

The purity of GHBA obtained by hydrolysis of GBL was determined by HPLC. For this purpose, HPLC system composed of UHPLC chromatograph (UltiMate 3000, Dionex, Sunnyvale, CA, USA), a Kinetex C18 column (4.6 × 100 mm, 2.6 μm; Phenomenex, USA) and PDA detector working in the range 190—400 nm was used. Mobile phase A was 25 mM formic acid in water; mobile phase B was 25 mM formic acid in acetonitrile. The gradient program started at 1% B increasing to 95% for 47 min, and ended with isocratic elution (95% B) for 3 min. The total run time was 50 min at the mobile phase flow rate 0.4 mL/min.

### GC with flame ionization detection (GC-FID) measurements

GC-FID model GC-2010 (Shimadzu, Kyoto, Japan) was used for quantitative analysis of DCM solution of GHBA standard, GHBA derivatives (GHBA-2TMS, GHBA-Me, cyclizated GHBA) and GBL standard. One μL samples were injected by an AOC-20i type autosampler into a ZB5-MSi fused-silica capillary column (30 m × 0.25 mm i.d., 0.25 μm film thickness) (Phenomenex, USA). Separation conditions were the following: carrier gas-hydrogen (grade 5.0); flow rate—1.0 mL/min; split injection mode with split ratio of 7:1. The temperature program involved: initial temperature 60 °C held for 3 min; temperature increase to 300 °C (at a rate of 10 °C/min) and maintained for 3 min. The chromatographic separations were performed at 300 °C.

The same equipment and chromatographic conditions were employed to investigate the effect of injector temperature on the signal intensity of GHBA and GBL, which were introduced in equivalent amounts. The only variable parameter in this case was the injection temperature, which was varied from 125 °C to 325 °C in 25 °C increments.

### GC–MS measurements

The same samples as above were analyzed with the use of GC–MS system. Gas chromatograph hyphenated with a triple quadrupole tandem mass spectrometer detector (GCMS-TQ8040; Shimadzu, Kyoto, Japan) was used in this case. GC–MS separation conditions were the same as those in GC-FID analysis. The injection temperature was the only difference. GC–MS analysis was performed only at one injection temperature—300 °C.

As to mass spectrometer parameters they were as follows: normalized electron energy of 70 eV; ion source temperature 225 °C. MS detector operated in Q3-SCAN mode with range *m/z* 30—350.

### GC–MS/MS measurements

The same as above equipment and the same working conditions were used for quantitative estimation of GHBA and GBL in extracts from not spiked and spiked plasma samples and in not spiked and spiked urine, wine, beer and orange juice samples. In this case, however, MRM mode of MS detector was applied.

Characteristic multiple reaction monitoring (MRM) transitions and optimal collision energies (CE) for GHBA, GBL, GHBA-2TMS, and est-Me-GHBA were determined using DCM solutions of GHBA and GBL standards (see Fig. 4SA), as well as post-derivatization mixtures containing GHBA-2TMS and est-Me-GHBA (see Fig. 4SB and C, respectively).

### Fourier transformed infra-red (FTIR) spectroscopy

As is known, hydroxycarboxylic acids can undergo not only cyclization to lactones, but also polycondensation to non-volatile high molecular weight compounds [21]. The formation of GHBA polycondensation products in the GC injector may also be the cause of the decrease in its GC signal. In order to confirm or exclude the possible polycondensation of GHBA in the GC injector, an FTIR spectroscopy of the dry extract from the glass wool constituting the filling of the injector liner, to which DCM solution of GHBA had been previously injected multiple times, was performed. The temperature of GC-FID injector—300 °C. The dry extract analyzed by FTIR spectroscopy was obtained by evaporating the dichloromethane extract from the glass wool under a stream of nitrogen.

The infrared (IR) spectrum of the examined dry extract was registered in the wavenumber range from 4000 to 400 cm^−1^ using a Nicolet™iS50 FTIR spectrometer (Thermo Fisher Scientific) with the attenuated total reflection (ATR) crystal.

The analogous experiment as described above was performed after multiple injections of GBL solution.

### Method validation

The possibilities of applying GHBA cyclization to enhance its GC signal were presented in this work employing biological and food samples. The validation parameters of GHBA quantitation with the preliminary GHBA cyclization were estimated using GHBA solutions in DCM and GHBA spiked plasma samples i.e. samples which can be directly injected to GC equipment and samples which require the employment of sample preparation procedure before their GC analysis. The validation parameters are gathered in Table 1S (see supplementary materials).

The methods were validated for linearity, limit of detection (LOD), limit of quantification (LOQ), and both intra- and inter-day precision and accuracy. To assess linearity, five replicate analytical procedures were performed at each concentration level. The LOD and LOQ were determined based on signal-to-noise ratios of 3 and 10, respectively.

Intra- and inter-day precision and accuracy were evaluated through statistical analysis of quantitative results obtained on the same day and over three different days, using five independent samples containing the tested compounds (1, 5, 10, and 25 µg/mL of GHBA, est-Me-GHBA, GBHA-2TMS and Cyclo-GHBA/GBL standard solutions in DCM and plasma samples).

Linearity was assessed using the least squares method and expressed as the coefficient of determination (R^2^).

To prepare calibration plots, DCM solutions of GHBA, est-Me-GHBA, GBHA-2TMS and Cyclo-GHBA/GBL and plasma samples spiked with these analytes were used. The concentration levels of the target analytes in the used samples were as follows:

- 0.1, 0.5, 2.5, 10 and 25 µg/mL for est-Me-GHBA and GBHA-2TMS,
- 0.01, 0.05, 0.25, 1, 2.5, and 10 µg/mL for GHBA and Cyclo-GHBA/GBL (calibration level for the low range), and
- 10, 25, 50, 100, and 250 µg/mL for GHBA and Cyclo-GHBA/GBL (calibration level for the high range).

The samples were prepared in three replicates.

### Statistical analysis

The experimental data in individual diagrams are presented as the mean values of three independent measurements (n = 3) ± SD. Appropriate experimental data were compared using analysis of variance (ANOVA). Differences in the studied groups were considered significant for p ≤ 0.05 and F_crit_ < F_exp_.

## Results

**Fig. 1:**
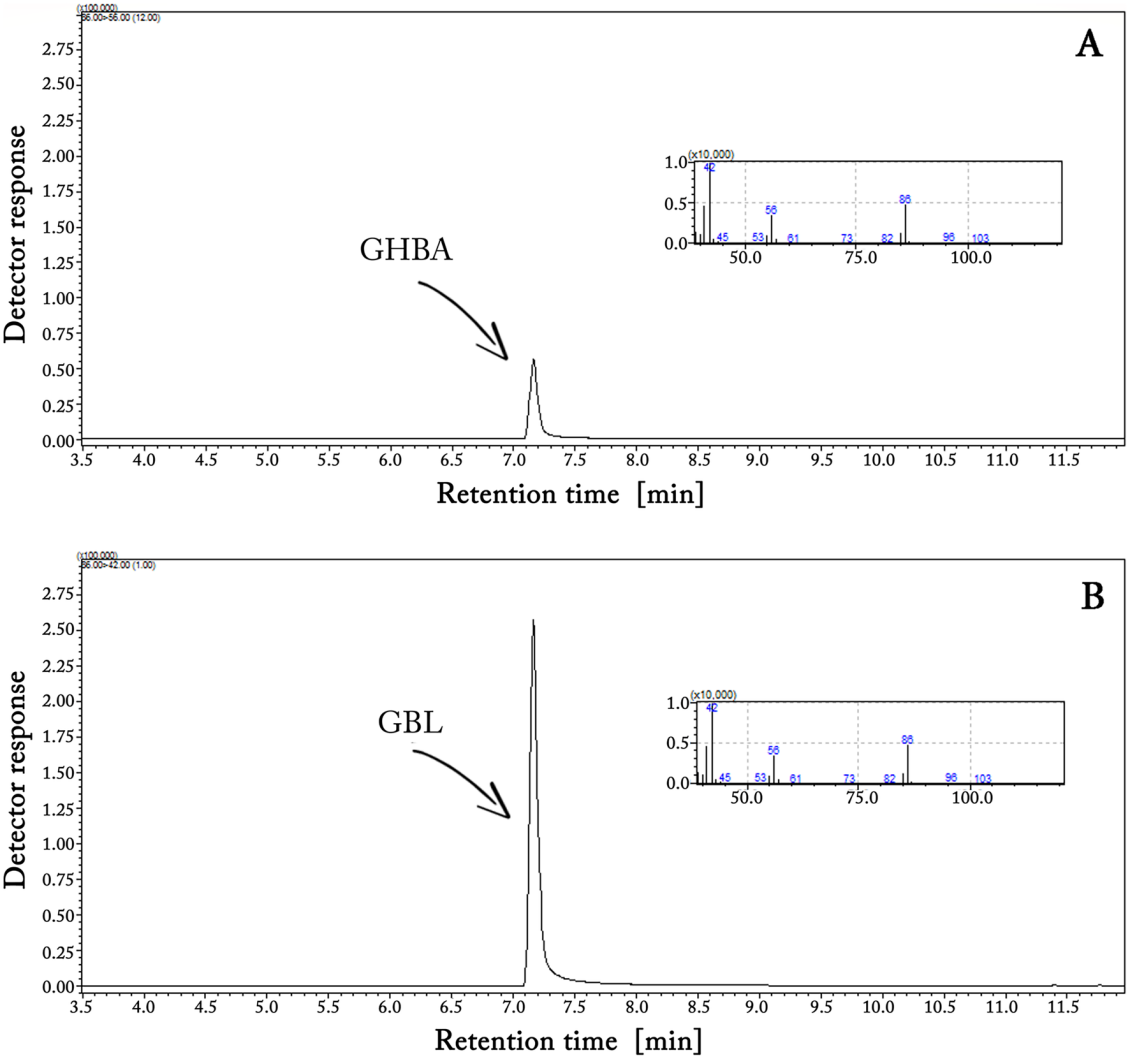
Exemplary GC–MS chromatograms (in total ion chromatogram (TIC) mode) of GHBA (**A**) and GBL (**B**) solutions in DCM, along with their MS spectra. In both cases, equivalent molar concentrations of the substances were injected

Figure 1 shows GC–MS chromatograms (in total ion chromatogram (TIC) mode) for GHBA (A) and GBL (B) solutions in DCM at concentrations of 0.1 and 0.083 mg/mL, respectively, and their corresponding MS spectra. The concentrations of individual substances were selected in such a way that equimolar amounts were introduced into the GC injector. The clearly lower GHBA peak in relation to GBL suggests that the former undergoes decomposition in the high-temperature GC injector.

**Fig. 2:**
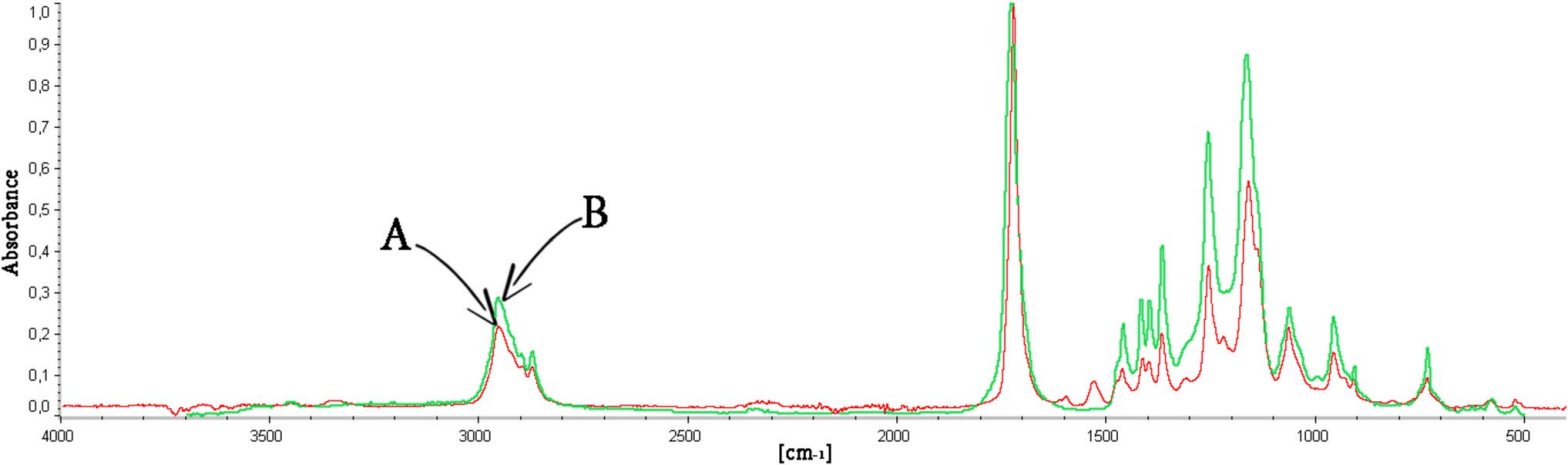
FTIR spectrum of: **A** the substance extracted from the glass wool filling the GC injector liner (injection temperature 300 °C) after 150 injections of GHBA solution in DCM (250 mg/mL), and **B** Morester 942 (the name of polyester which, according to the IR library data, shows the highest degree of matching with the extracted substance)

Figure 2 shows the FTIR spectrum of:

- the substance extracted from the glass wool filling the GC injector liner (injection temperature 300 °C) after 150 injections of GHBA solution in DCM (250 mg/mL), and
- the substance which, according to the IR library data, shows the highest degree of matching with the extracted substance—i.e. with polyester plasticizer commercially named as Morester942.

**Fig. 3:**
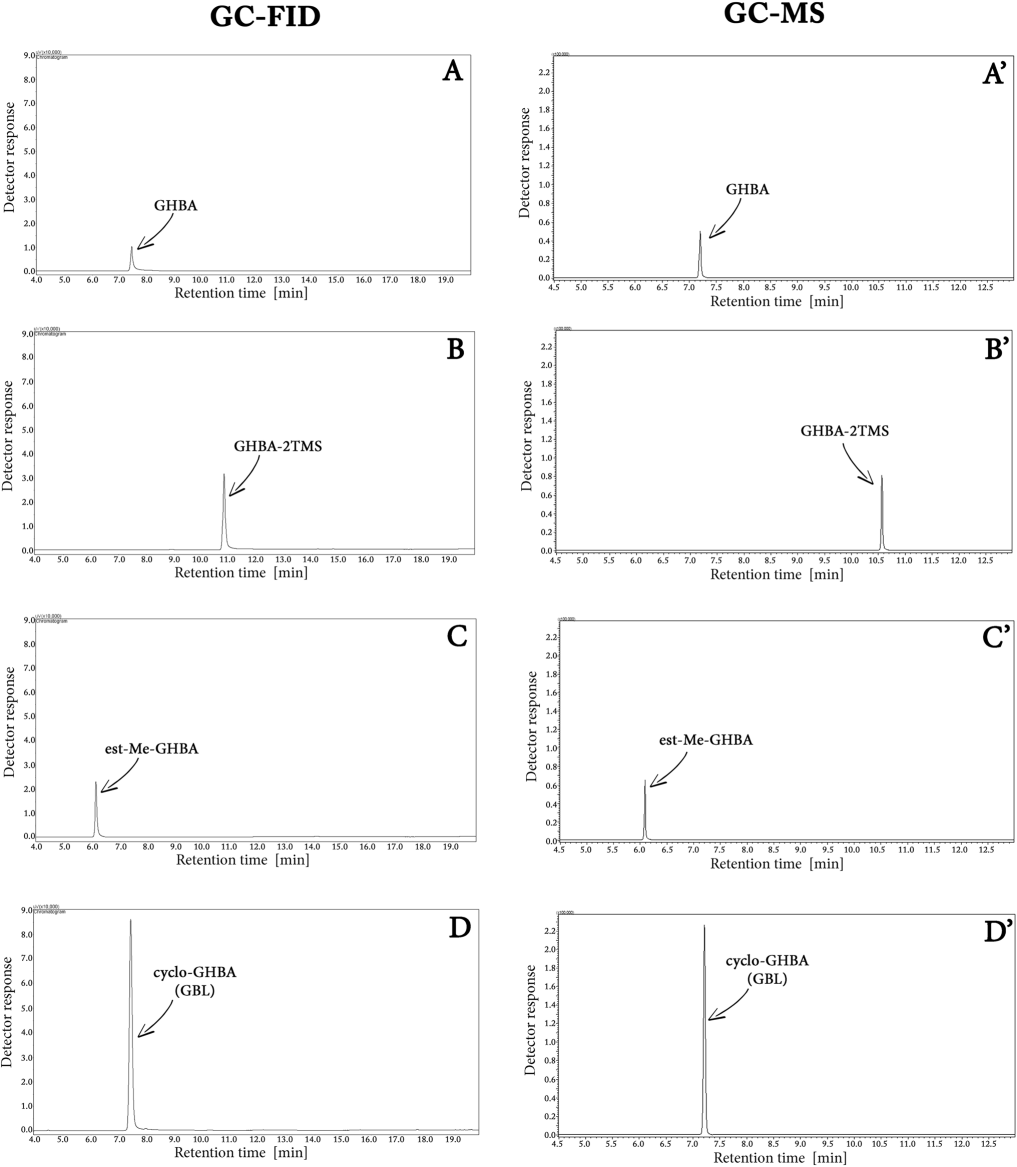
GC-FID and GC–MS (in TIC mode) chromatograms of a GHBA sample (0.1 mg/mL in DCM): not subjected to the derivatization process (**A**, **A’**), silylated to form GHBA-2TMS (**B**, **B’**), methylated to form ester GHBA derivative (est-Me-GHBA) (**C**, **C’**), cyclized to form cyclo-GHBA, i.e. GBL (**D**, **D’**)

The classic way to increase the chromatographic signal of the analyzed substance is its derivatization. Derivatization is also a method to prevent the decomposition of the substance in the high-temperature GC injector. Figure 3 shows GC-FID and GC–MS chromatograms (in TIC) of a GHBA sample (0.1 mg/mL in DCM), which:

- was not subjected to the derivatization process (A, A’, respectively)
- was silylated to form GHBA-2TMS (B, B’, respectively)
- was methylated to form ester GHBA derivative (est-Me-GHBA) (C, C’, respectively) and
- was cyclized to form cyclo-GHBA, i.e. GBL (D, D’, respectively).

The figure indicates that although classical derivatization methods, silylation and methylation, increase the GHBA signal in both GC-FID and GC–MS systems, a much greater increase in the signal is observed in the case of cyclization of GHBA to GBL.

In order to check whether GBL itself does not polymerize under high-temperature injection conditions, its solution in DCM (250 mg/mL) was injected 150 times to the GC system, then the glass wool filling the GC injector liner was removed, extracted and the obtained extract was examined using FTIR. The result of this experiment is presented in Fig. 5S (see supplementary materials). It shows that GBL does not form polyester in the GC liner, and if it does, it is in an amount lower than the FTIR detection limit.

**Fig. 4:**
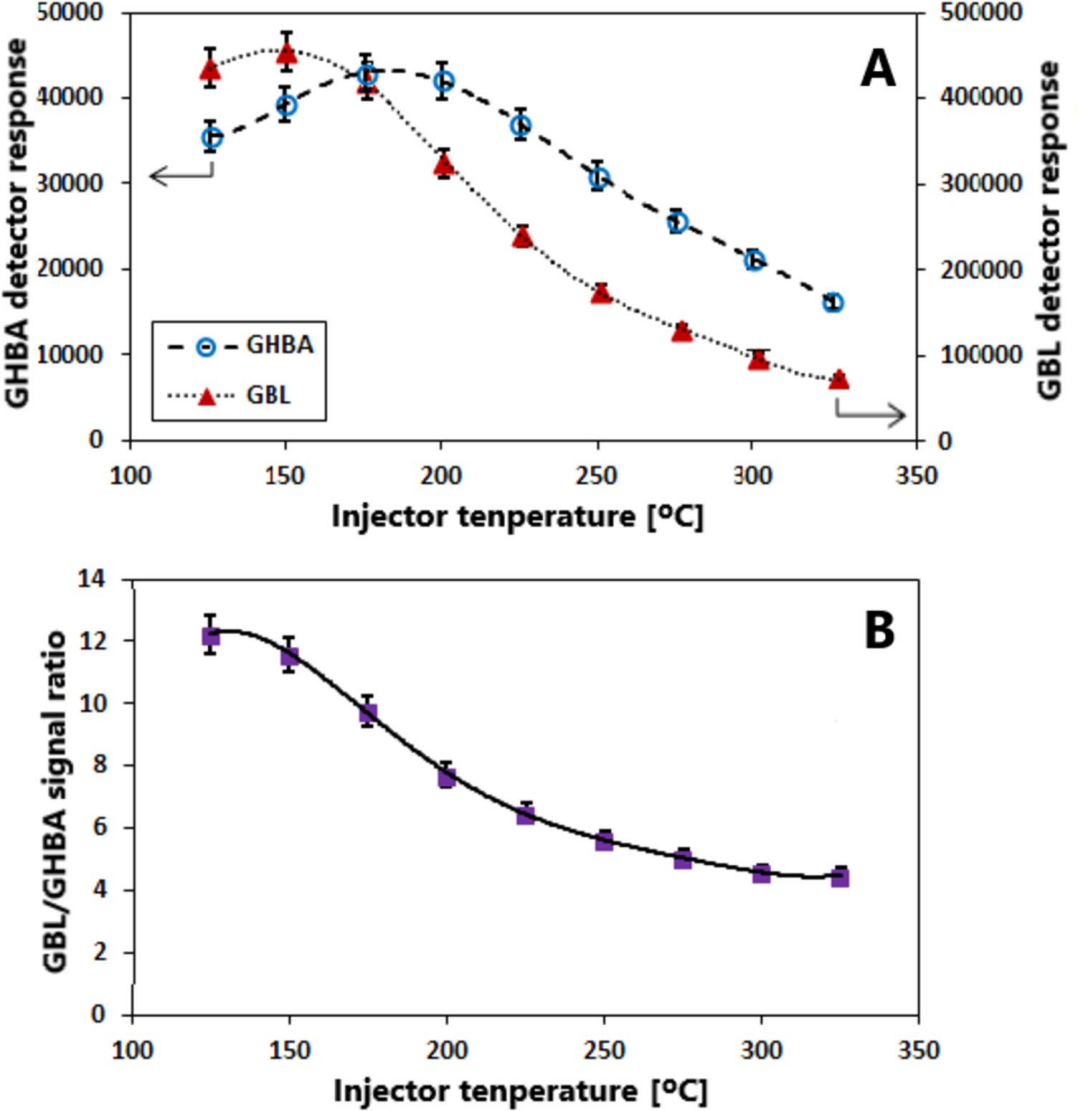
The effect of injector temperature on: the GC signal intensities of GHBA and GBL (**A**) and the ratio of GHBA and GBL signal responses (**B**). Equivalent concentrations of GHBA and GBL were injected (1 mg/mL and 0.83 mg/mL, respectively)

**Fig. 5:**
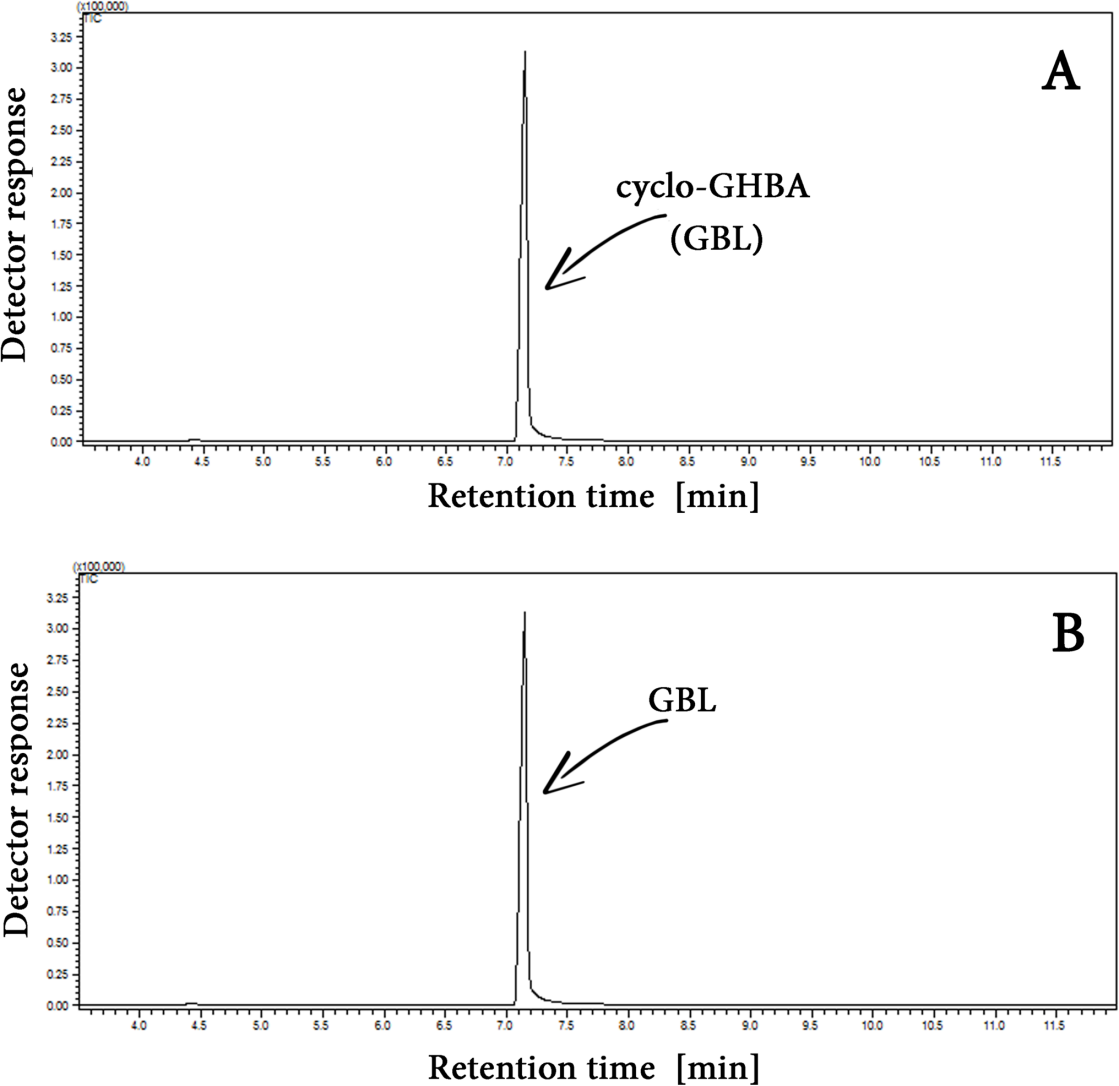
GC–MS chromatograms (in TIC mode) of (**A**) GHBA sample (0.1 mg/mL in DCM) after the cyclization procedure and of (**B**) GBL solution in DCM (0.083 mg/mL)

Figure 4A presents the effect of injector temperature on the GC signal intensities of GHBA and GBL, which were injected at equivalent concentrations (1 mg/mL and 0.83 mg/mL, respectively), while Fig. 4B illustrates the influence of temperature on the ratio of their signal responses. These findings are consistent with the data shown in Fig. 5S—see the analysis and comparison of the data in Figs. 5S and 4 in the Discussion section.

In view of the facts indicating that cyclization of GHBA is a much more effective way of increasing the GC-FID/GC–MS signal of this compound than its silylation and methylation, studies were carried out to determine the efficiency of the GHBA to GBL cyclization reaction at the sample preparation stage. Their results indicate that the process proceeds with an efficiency of over 99%.—compare the example GC–MS signals (in TIC) for GHBA solution in DCM (0.1 mg/mL) after the cyclization process and GBL solution in DCM (0.083 mg/mL) (equimolar concentrations) presented in Fig. 5.

**Fig. 6:**
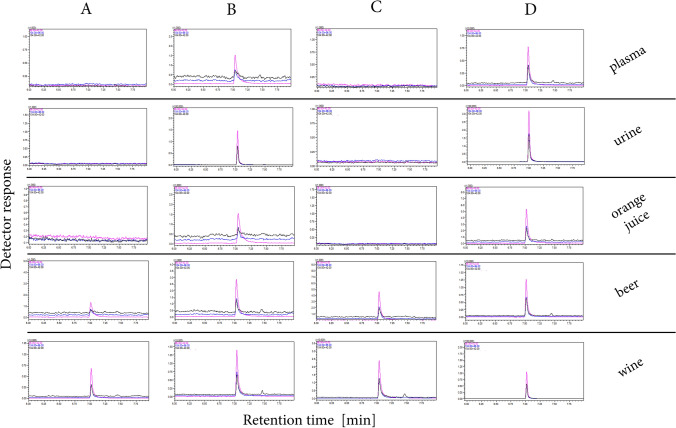
MRM chromatograms of plasma, urine, orange juice, beer and wine samples: blank samples before (**A**) and after cyclization process (**C**); GHBA spiked samples before (**B**) and after cyclization process (**D**). Injection temperature − 300 °C

The practical aspect of using GHBA cyclization to increase the sensitivity of its GC analysis is presented in Fig. 6. It shows GC–MS/MS chromatograms in MRM mode for plasma, urine, orange juice, beer and wine samples spiked with GHBA.

The validation data for GHBA, GHBA-2TMS, est-Me-GHBA and cyclo GHBA (i.e. GBL) are gathered in Table 1S.

In order to obtain the data presented in Figs. 1, 3, 4, 5, 6, 5S and Table 1S, it was necessary to synthesize the standards GHBA, GHBA-2TMS, and est-Me-GHBA. These compounds were prepared according to the procedures described in the Experimental section (see Methods) and were subsequently analyzed chromatographically. The chromatograms of the resulting standard solutions are shown in Figures S1, S2, S3. As shown in Figure S1, the obtained GHBA does not contain the starting substrate (i.e., GBL) or any detectable impurities, or they are present below the detection limit. The chromatogram presented in Figure S2 demonstrates that the solution obtained after silylation of GHBA, apart from the components of the derivatization mixture, contains only GHBA-2TMS. A similar conclusion can be drawn from Figure S3, which refers to the GHBA solution subjected to methylation. It confirms the complete conversion of GHBA to est-Me-GHBA. Considering the nearly 100% yield of the synthesis of the above compounds and the known concentrations of the starting substrates (GBL or GHBA), these solutions were used in the study as standard solutions.

The same solutions were utilized for the optimization of collision energies in the multiple reaction monitoring (MRM) transitions of the compounds under study. Three MRM transitions (*m/z* =  > *m/z*) of the highest intensity were selected for data presented in Fig. 6 and Table 1S:

104 > 86 (CE = 9 eV), 104 > 42 (CE = 6 eV) and **86 > 42** (CE = 6 eV) for GBL/GHBA,

248 > 233 (CE = 15 eV), 248 > 73 (CE = 12 eV) and **233 > 147** (CE = 12 eV) for GHBA-2TMS, 118 > 88 (CE = 12 eV), **118 > 74** (CE = 12 eV) and 88 > 43 (CE = 9 eV) for est-Me-GHBA, MRM transitions selected for quantification of individual analytes were bolded and underlined. The optimizations of collision energies for MRM transitions of GBL, GHBA, GHBA-2TMS and est-Me-GHBA are provided in Fig. 4S.

Figure 6S compares the recovery of GBL, generated via GHBA cyclization in plasma and water samples, using PTSA, as recommended in this study, and H_2_SO_4_, the reagent most commonly employed for this purpose [28]. Recovery was determined by relating the GBL signal obtained during GC–MS analysis of the examined extracts (see “*PTSA vs. H*_*2*_*SO*_*4*_* as cyclization agents for GHBA present in plasma and water samples*” in the experimental section) to the corresponding signal from its standard solution. The concentration of GBL in its standard solution was 0.664 µg/mL, which corresponds to a 100% conversion of GHBA to GBL and a 100% recovery of GBL in the extraction process.

## Discussion

According to the literature, there are established procedures recommending the cyclization of GHBA into GBL for the purpose of GHBA determination using GC techniques [29–34]. However, it is worth considering the benefits of such a transformation and its impact on the sensitivity of GHBA analysis compared to that achieved using conventional derivatization methods, i.e. GHBA silylation and methylation.

Figure 1 demonstrates that GHBA undergoes conversion to its lactone form (GBL) in the high-temperature GC injector during analysis. This is evidenced by the identical mass spectra and retention times observed for both GHBA and GBL. The difference in the signal size of both compounds, which were dosed in equimolar amounts in this experiment, suggests that GHBA not only undergoes cyclization in the GC injector but probably also undergoes partial conversion to compounds with lower molecular weights or non-volatile compounds. The lack of additional peaks in the GHBA chromatogram (see Fig. 1A) and the presence in the GHBA molecule of functional groups capable of mutual reaction not only towards the formation of cyclic GBL but also towards the formation of linear polyesters indicate the latter possibility. It is confirmed by the results of spectroscopic studies of the extract from glass wool filling the GC injector liner (see Fig. 2). Thus, the lower GC signal for GHBA than for GBL is due to the partial polycondensation of the former to a non-volatile polyester that takes place in the GC injector. Thus, the data presented in Figs. 1 and 2 support the justification for the cyclization of GHBA into GBL prior to the analysis of the former, as proposed in [29–34].

The most commonly used method of increasing the GC signal of the analyzed substance is its derivatization. It leads to modification of the functional groups of the analyzed molecule, which are capable of specific interaction with the stationary phase of the chromatographic column. In the case of GHBA, the OH and COOH groups capable of specific interaction with the stationary phase also have the ability to react with each other, as a result of which, among others, polyester is formed. The formation of polyester from GHBA can be prevented by blocking its functional groups by reaction with the derivatizing reagent at the stage of sample preparation for analysis, e.g. by its silylation or methylation. It should be noted that during the cyclization process of GHBA to GBL, the hydroxyl and carboxyl groups—responsible for specific interactions with the stationary phase and for the polycondensation reaction—are also blocked. Figure 3 chromatograms clearly demonstrate that all of the aforementioned methods lead to an enhancement of the GHBA signal in both GC-FID and GC–MS (TIC) analyses. In the case of silylation and methylation of GHBA, the increase in the signal is 1.5 and 1.3 times, respectively, while in the case of cyclization of GHBA, the signal from this compound increases as much as 4.6 times. This 4.6-fold increase in the GHBA signal caused by its cyclization to GBL at the stage of sample preparation for GC-FID or GC–MS analysis is associated with the elimination of losses of this compound, which are the effect of its polycondensation in the GC injector. This is confirmed by the results of FTIR analysis of the glass wool extract extracted from the GC injector liner after 150 injections of GBL solution in DCM (250 mg/mL) presented in Fig. 5S. They show that GBL, unlike GHBA, does not form polyester in the GC liner, and if it does, it is in an amount lower than the FTIR detection limit.

The results presented in Fig. 4 complement the data shown in Fig. 5S by illustrating the effect of injection temperature on the GC-FID signal intensity of GHBA and GBL. In this experiment, equimolar amounts of both compounds were introduced into the injector. As demonstrated in Fig. 4, throughout the examined temperature range, the GC-FID signal intensity for GHBA is significantly lower than that for GBL (note the differences in the axes’ scales). This observation is consistent with the previously presented evidence indicating that, in the high-temperature GC injector, a portion of GHBA is converted into a non-volatile polyester, while the remaining fraction is cyclized into GBL. The relationships depicted in Fig. 4 also suggest that increasing the injection temperature initially causes a slight increase, followed by a decrease, in the signal intensity of both compounds. However, it should be noted that in the case of GHBA, these signal variations are markedly smaller than those observed for GBL (again, consider the scales used in the plots). This indicates that, within the studied temperature range, the degree of GHBA transformation is affected to a significantly lesser extent by injector temperature compared to GBL. This implies that, across the entire investigated temperature range, a considerable but relatively less variable percentage of GHBA undergoes cyclization. At this point, it is essential to address the question of how increasing the injection temperature influences the ratio of signal intensities of the two compounds. The answer is provided in Fig. 4B. On the one hand, it shows that the extent of GHBA transformation is greater than that of GBL over the entire temperature range. On the other hand, it demonstrates that the GBL/GHBA signal ratio is consistently greater than 1. This latter observation confirms that the preliminary cyclization of GHBA to GBL invariably results in an increase in the GBL signal, regardless of injector temperature—with a more pronounced increase at lower injection temperatures (150–200 °C) and a less substantial increase at higher temperatures (250–325 °C). It should be noted that the chromatograms presented in Fig. 3 were obtained at an injector temperature of 300 °C, which falls within the second of the aforementioned temperature ranges.

Considering the above discussion and the fact that the conversion rate of GHBA to GBL during sample preparation approaches 100% (see Fig. 5), the proposed method for enhancing the GHBA signal to improve the sensitivity of its analysis via gas chromatography is well justified and merits recommendation. Although procedures recommending the conversion of GHBA to GBL for the purpose of GHBA quantification using GC techniques are well-documented in the literature [29–34], they typically involve the use of H₂SO₄ or other strong acids (e.g., HCl, HClO₄) to induce this process. However, when applied to protein-rich samples such as blood or plasma, these reagents also act as protein-precipitating agents. As demonstrated in the literature [35–37], protein precipitation can lead to co-precipitation of analytes, resulting in their loss during sample preparation. Support for the above statement regarding GHBA is provided by the results presented in Fig. 6SA. As shown, the use of H_2_SO_4_ for the cyclization of GHBA in plasma samples results in a lower recovery of the formed GBL compared to the use of PTSA (F_exp_ > F_crit_). This can be attributed to the formation of a significant amount of denatured protein precipitate in the plasma sample during the cyclization of GHBA using H_2_SO_4_. Based on the results in Fig. 6SA, it cannot be excluded that the use of H_2_SO_4_ in the GHBA cyclization process also promotes its polycondensation, thereby limiting the complete conversion to GBL. However, since the recovery of GBL from aqueous GHBA solutions is nearly 100% (see Fig. 6SB), regardless of the cyclization agent used (H_2_SO_4_ or PTSA), the latter hypothesis for GHBA loss appears unlikely. The GHBA cyclization procedure to GBL proposed in this study does not result in analyte loss. The recovery of GHBA in the form of GBL is quantitative (100%).

The practical utility of the signal enhancement of GHBA by its cyclization is presented in Fig. 6 showing MRM chromatograms of plasma, urine, orange juice, beer and wine samples spiked and not spiked with GHBA. Classical extraction and extraction with the GHBA cyclization process were used as sample preparation methods in these experiments. The presence of GHBA/GBL peaks in blank samples of beer and wine is not surprising (see Fig. 6A and C), as these compounds are native constituents of such beverages. The chromatograms indicate that the novel procedure of GHBA cyclization is applicable in the analytical procedures of real samples—the cyclization process causes a multiple increase of the GHBA signal.

## Conclusions

The performed experiments indicate that the low response of MS and FID detectors towards GHBA results from its partial polycondensation to polyester, which occurs in GC injector. The most commonly used method to increase the sensitivity of chromatographic analysis of the compounds studied is their preliminary derivatization. The presented results show, however, that although the increase in GHBA signal can be achieved through its silylation and/or methylation, the largest increase is observed in the case of its cyclization to GBL during the sample preparation process. Although the conversion of GHBA to GBL for GC-based quantification is well-documented and typically involves strong acids such as H₂SO₄, in protein-rich samples (e.g., blood or plasma) these reagents may cause analyte loss due to co-precipitation with proteins. Moreover, the acidic environment can promote GHBA polycondensation, leading to further analyte loss.

The procedure of GHBA cyclization described in this paper is notably straightforward and highly effective (nearly 100% analyte recovery) and allows for the quantification of this hydroxycarboxylic acid in various matrices such as plasma, urine, wine, beer, and orange juice samples. Moreover, the proposed procedure does not lead to as rapid contamination of the GC injector and MS filaments as is observed when using the silylation of compounds (including GHBA) to enhance their signal.

The increase in the GHBA signal presented in the work, resulting from its initial cyclization, encourages research on the influence of cyclization of other hydroxycarboxylic acids on the change in their GC signal.

## Supplementary Information

Below is the link to the electronic supplementary material.

Supplementary file1 (TIF 85 KB) Fig. 1S HPLC chromatograms (205 nm) of GHBA standard (A), GBL standard (B) and GHBA obtained by GBL hydrolysis (C)

Supplementary file2 (TIF 1705 KB) Fig. 2S GC-FID chromatograms of silylation mixture (A), GHBA standard (B) and post-reaction mixture obtained after GHBA silylation (C)

Supplementary file3 (TIF 1527 KB) Fig. 3S GC-FID chromatograms of methylation mixture (A), GHBA standard (B) and post-reaction mixture obtained after GHBA methylation (C)

Supplementary file4 (TIF 2321 KB) Fig. 4S Collision energies optimization (CE) for quantitative MRM transitions of: GHBA/GBL (A), GHBA-2TMS (B), and est-Me-GHBA (C)

Supplementary file5 (TIF 2321 KB)

Supplementary file6 (TIF 59 KB) Fig. 5S FTIR spectrum of: (A) the substance extracted from the glass wool filling the GC injector liner (injection temperature 300 oC) after 150 injections of GHBA solution in DCM (250 mg/mL), and (B) the substance extracted from the glass wool filling the GC injector liner (injection temperature 300 oC) after 150 injections of GBL solution in DCM (250 mg/mL)

Supplementary file7 (TIF 10 KB) Fig. 6S The recovery of GBL from plasma (A) and water (B) samples after GHBA cyclization using PTSA (green bars) and H2SO4 (red bars)

Supplementary file8 (DOCX 17 KB)

## Data Availability

All data generated or analyzed during the study are included in the present article.
