# Supplementary figures and images for: Cyclization of γ-hydroxybutyric acid (GHBA) as a strategy to enhance its signal in gas chromatography analysis
Source: Forensic Toxicol. 2025 Sep 13;44(1):107–19. doi: 10.1007/s11419-025-00738-z (PMC12858631; doi:10.1007/s11419-025-00738-z)

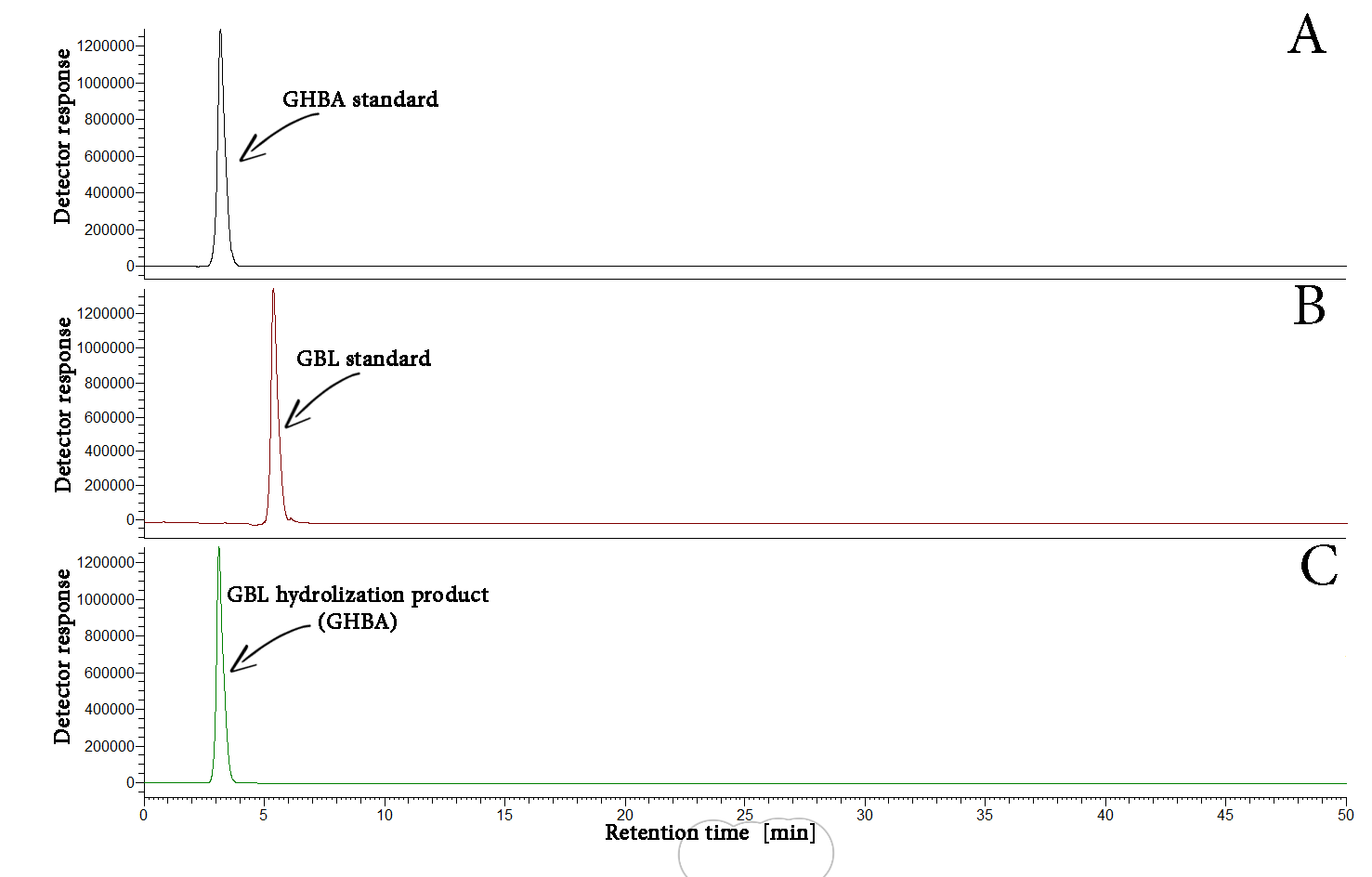

Supplement: Supplementary file 1 — Supplementary file1 (TIF 85 KB) Fig. 1S HPLC chromatograms (205 nm) of GHBA standard (A), GBL standard (B) and GHBA obtained by GBL hydrolysis (C) [file 11419_2025_738_MOESM1_ESM.tif]

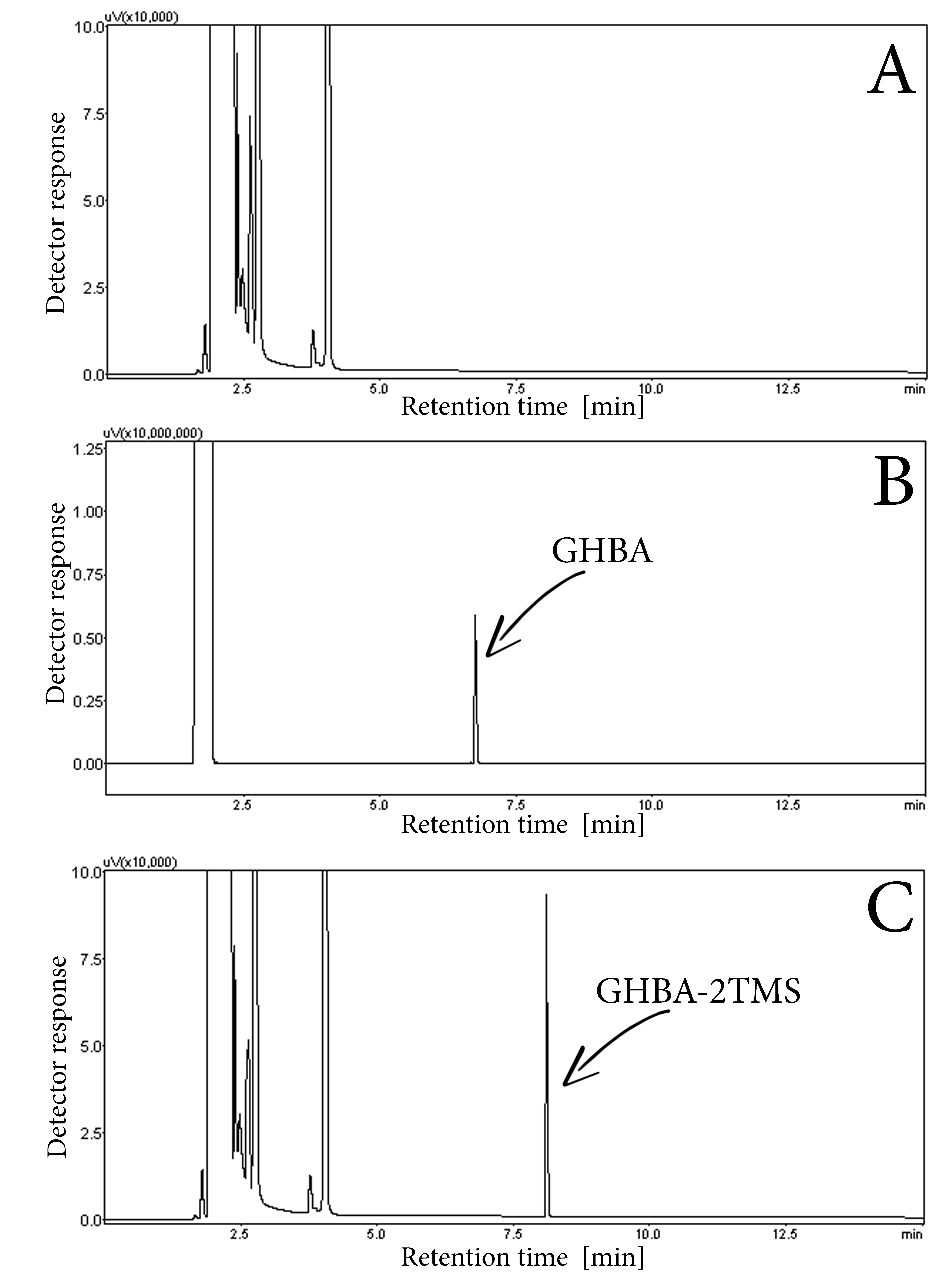

Supplement: Supplementary file 2 — Supplementary file2 (TIF 1705 KB) Fig. 2S GC-FID chromatograms of silylation mixture (A), GHBA standard (B) and post-reaction mixture obtained after GHBA silylation (C) [file 11419_2025_738_MOESM2_ESM.tif]

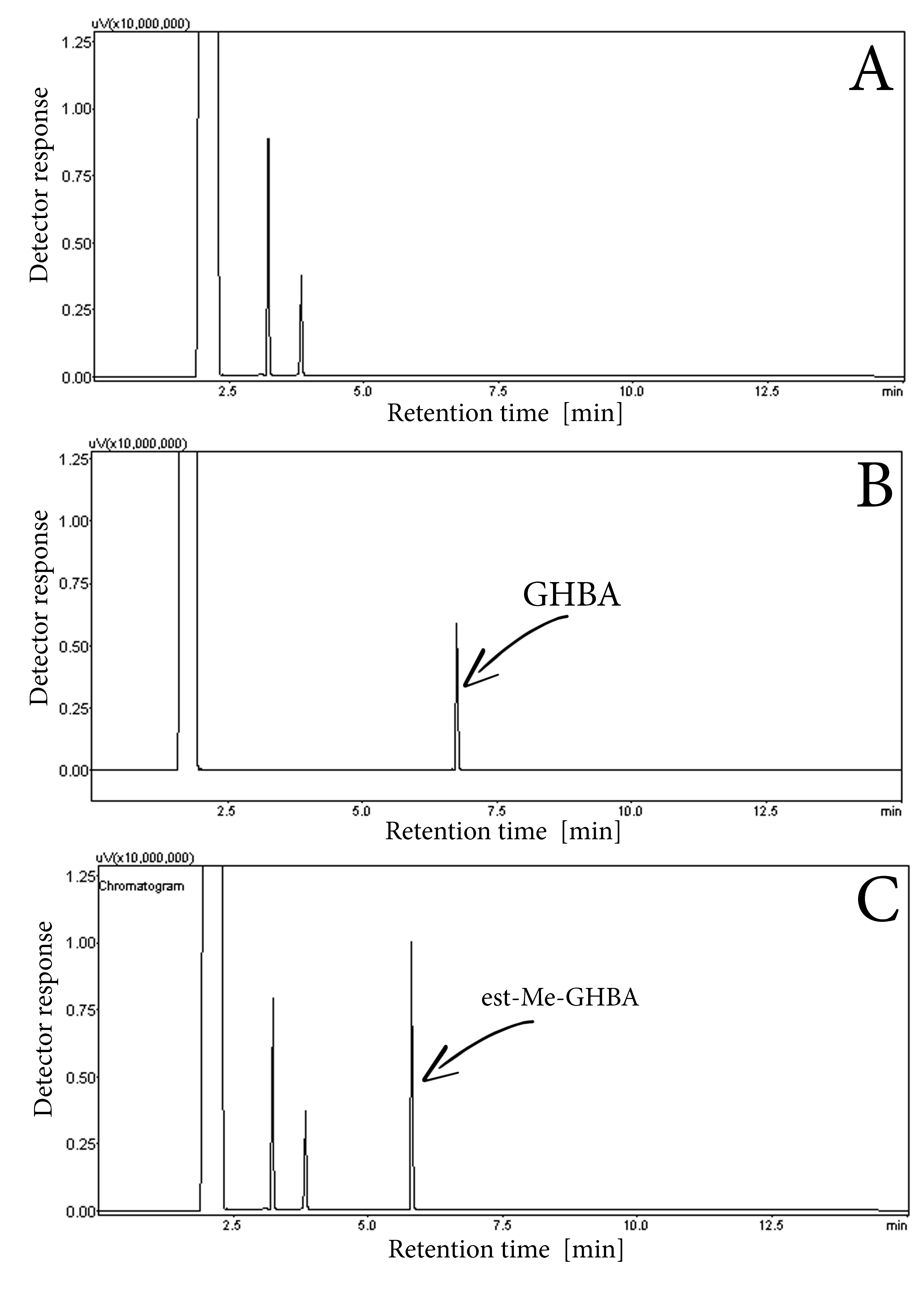

Supplement: Supplementary file 3 — Supplementary file3 (TIF 1527 KB) Fig. 3S GC-FID chromatograms of methylation mixture (A), GHBA standard (B) and post-reaction mixture obtained after GHBA methylation (C) [file 11419_2025_738_MOESM3_ESM.tif]

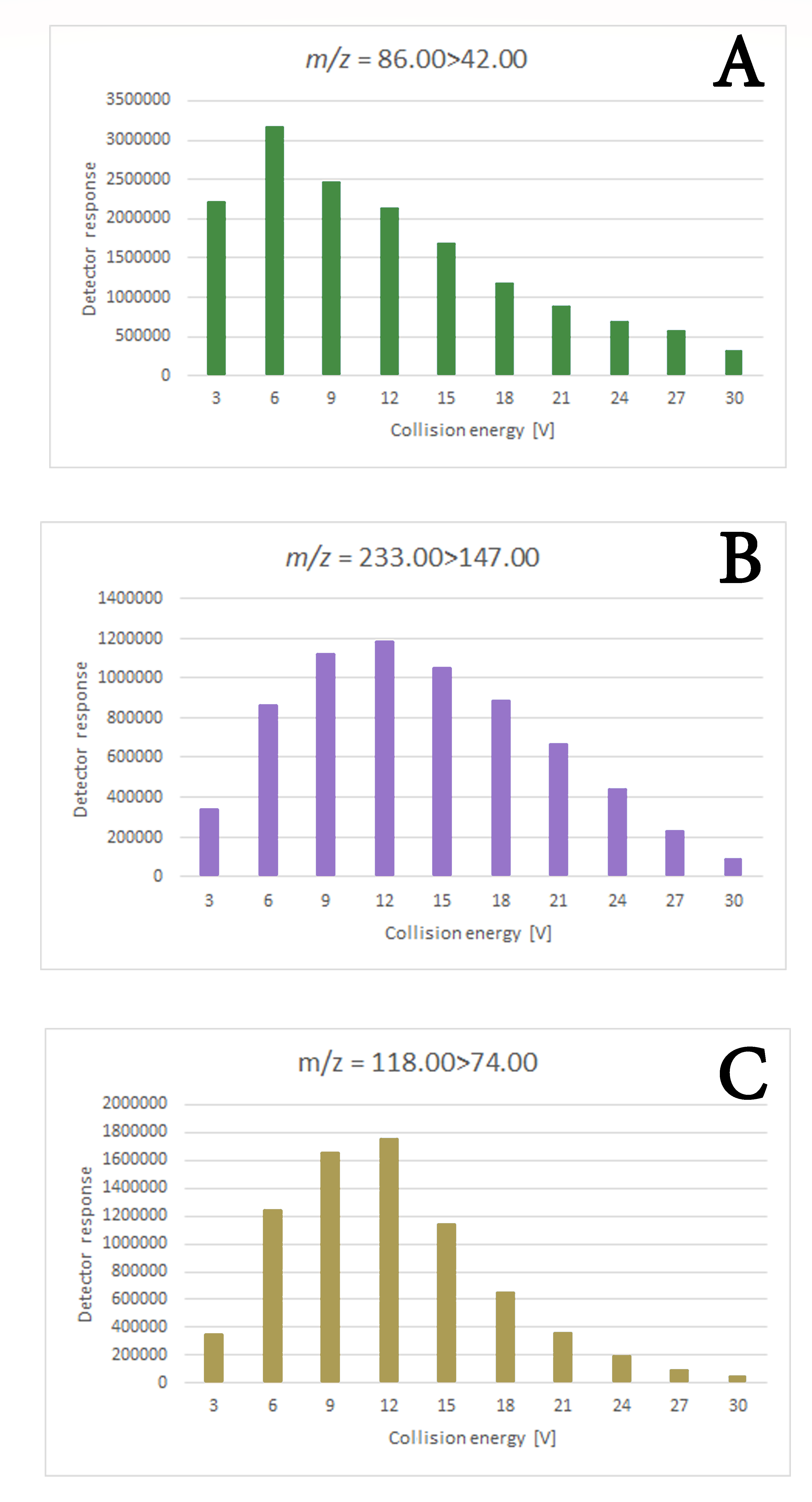

Supplement: Supplementary file 4 — Supplementary file4 (TIF 2321 KB) Fig. 4S Collision energies optimization (CE) for quantitative MRM transitions of: GHBA/GBL (A), GHBA-2TMS (B), and est-Me-GHBA (C) [file 11419_2025_738_MOESM4_ESM.tif]

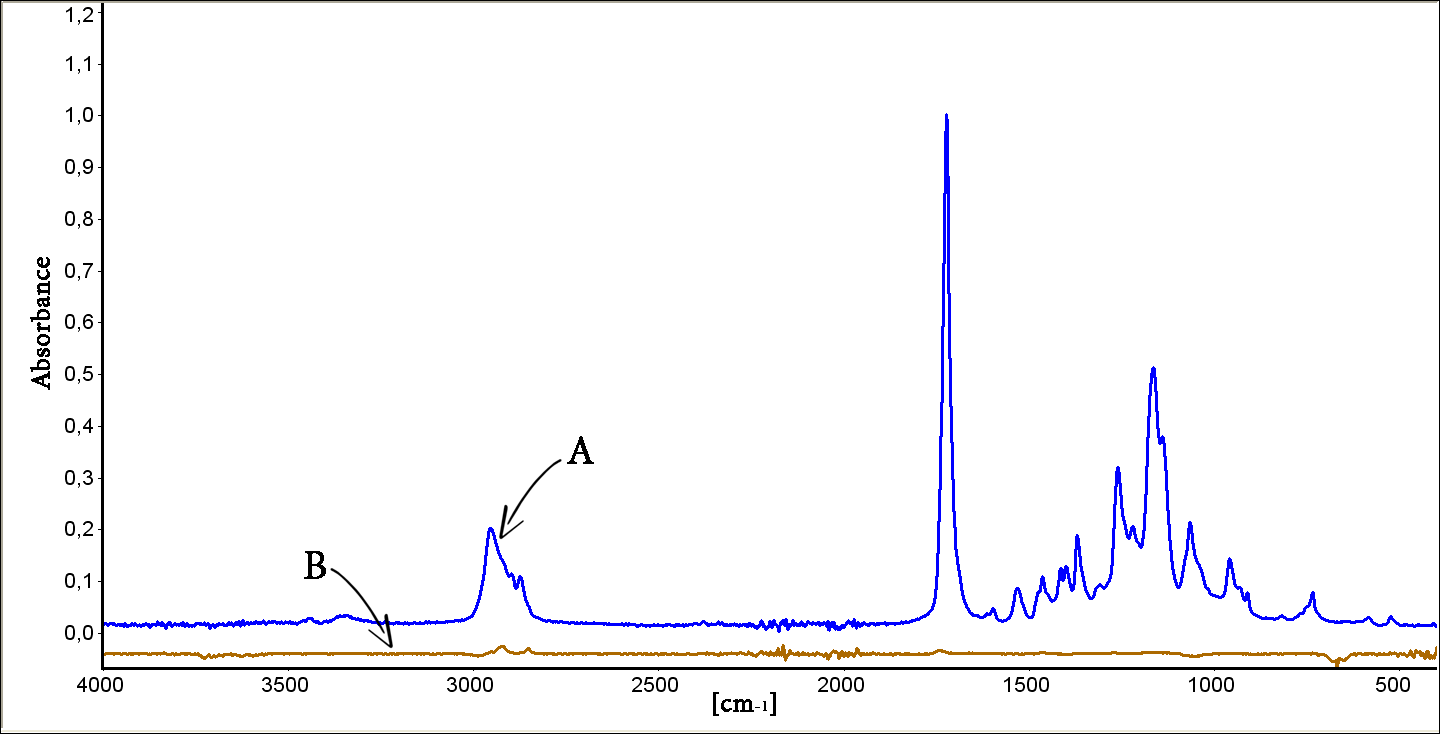

Supplement: Supplementary file 6 — Supplementary file6 (TIF 59 KB) Fig. 5S FTIR spectrum of: (A) the substance extracted from the glass wool filling the GC injector liner (injection temperature 300 oC) after 150 injections of GHBA solution in DCM (250 mg/mL), and (B) the substance extracted from the glass wool filling the GC injector liner (injection temperature 300 oC) after 150 injections of GBL solution in DCM (250 mg/mL) [file 11419_2025_738_MOESM6_ESM.tif]

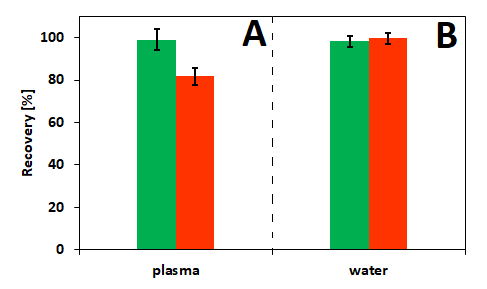

Supplement: Supplementary file 7 — Supplementary file7 (TIF 10 KB) Fig. 6S The recovery of GBL from plasma (A) and water (B) samples after GHBA cyclization using PTSA (green bars) and H2SO4 (red bars) [file 11419_2025_738_MOESM7_ESM.tif]
