# Supplementary material for: Cyclization of γ-hydroxybutyric acid (GHBA) as a strategy to enhance its signal in gas chromatography analysis
Source: Forensic Toxicol. 2025 Sep 13;44(1):107–19. doi: 10.1007/s11419-025-00738-z (PMC12858631; doi:10.1007/s11419-025-00738-z)
Supplement: Supplementary file 8 — Supplementary file8 (DOCX 17 KB) [file 11419_2025_738_MOESM8_ESM.docx]

**Table 1** Linearities, intra- and interday precisions and accuracies, limits of detection (LOD), limits of quantification (LOQ) of GHBA

est-Me-GHBA, GBHA-2TMS, cyclo-GHBA/GBL.

| **Tested parameter** | **Sample matrix** | | | | | | | |
| --- | --- | --- | --- | --- | --- | --- | --- | --- |
|  | **DCM solution** | | | | **Plasma** | | | |
|  | **Compound** | | | | | | | |
|  | **GHBA** | **est-Me-GHBA** | **GBHA-2TMS** | **Cyclo-GHBA/GBL** | **GHBA** | **est-Me-GHBA** | **GBHA-2TMS** | **Cyclo-GHBA/GBL** |
| **Linearity – calibration level for the low range**  **(R^2^)** | 0.9994 | 0.9985 | 0.9982 | 0.9990 | 0.9984 | 0.9971 | 0.9968 | 0.9983 |
| **Linearity - calibration level for the high range**  **(R^2^)** | 0.9996 | 0.9991 | 0.9987 | 0.9994 | 0.9991 | 0.9979 | 0.9977 | 0.9992 |
| **Intraday precision**  **(% RSD)** | 2.99 | 3.38 | 3.57 | 3.05 | 3.31 | 3.74 | 3.93 | 3.35 |
| **Interday precision**  **(% RSD)** | 3.62 | 4.01 | 4.37 | 3.72 | 3.98 | 4.47 | 4.83 | 4.11 |
| **Intraday accuracy**  **(%)** | 99.09 | 98.12 | 97.92 | 98.97 | 94.72 | 92.81 | 92.58 | 93.57 |
| **Interday accuracy**  **(%)** | 97.96 | 97.21 | 96.85 | 97.63 | 92.58 | 91.94 | 91.54 | 92.22 |
| **LOD**  **(µg/mL)** | 0.22 | 0.17 | 0.13 | 0.04 | 0.28 | 0.21 | 0.13 | 0.04 |
| **LOQ**  **(µg/mL)** | 0.75 | 0.56 | 0.42 | 0.13 | 0.94 | 0.70 | 0.46 | 0.13 |

R^2^ coefficient of determination, RSD relative standard deviation, LOD limit of detection, LOQ limit of quantification
